# Is autophagy induction by PARP inhibitors a target for therapeutic benefit?

**DOI:** 10.32604/or.2022.026459

**Published:** 2022-12-06

**Authors:** AHMED M. ELSHAZLY, TUONG VI V. NGUYEN, DAVID A. GEWIRTZ

**Affiliations:** 1 Department of Pharmacology and Toxicology, Virginia Commonwealth University, Massey Cancer Center, Richmond, VA, 23298, USA; 2 Department of Pharmacology and Toxicology, Faculty of Pharmacy, Kafrelsheikh University, Kafrelsheikh, 33516, Egypt

**Keywords:** Olaparib, Niraparib, Rucaparib, Talazoparib, Autophagy, Cytoprotective, Cytotoxic, Non-protective

## Abstract

PARP inhibitors have proven to be effective in conjunction with conventional therapeutics in the treatment of various solid as well as hematologic malignancies, particularly when the tumors are deficient in DNA repair pathways. However, as the case with other chemotherapeutic agents, their effectiveness is often compromised by the development of resistance. PARP inhibitors have consistently been reported to promote autophagy, a process that maintains cellular homeostasis and acts as an energy source by the degradation and reutilization of damaged subcellular organelles and proteins. Autophagy can exhibit different functional properties, the most prominent being cytoprotective. In addition, both cytotoxic and non-protective functions forms have also been identified. In this review, we explore the available literature regarding the different roles of autophagy in response to clinically-used PARP inhibitors, highlighting the possibility of targeting autophagy as an adjuvant therapy to potentially increase the effectiveness of PARP inhibition and to overcome the development of resistance.

## Introduction

This manuscript is one of a series of papers that explore the role of autophagy in the response to therapeutic modalities in tumor cells. Our previous publications covered radiation [1], cisplatin [2], microtubules poisons [3], Topoisomerase I inhibitors (under review) as well as hormonal therapies in ER positive breast cancer [4].

### PARP inhibitors

The poly (ADP-ribose) polymerases (PARP) are a family of enzymes that catalyses ADP-ribose transfer to target proteins, which is termed poly ADP-ribosylation [5]. The PARP family of enzymes involves many isoforms, having a vital role in various cellular processes, including cell proliferation and cell death [6]. Among the 18 isoforms within the PARP family, PARP1 and PARP2 are best known for their contributions towards DNA repair pathways.

Cellular DNA damage is induced by various cancer chemotherapeutic modalities, including ionizing radiation. Single-stranded DNA breaks (SSBs) promote PARP activation to facilitate base excision repair (BER) [7]. PARP are members of the base excision repair complex, which consists of DNA polymerase beta, DNA ligase III, as well as X-ray repair cross-complementing 1 **(**XRCC1) protein [8]. PARP detect and bind to DNA strand break sites via the DNA-binding domain, followed by the synthesis of poly (ADP) ribose (PAR) and its allocation to the targeted proteins. PAR allows for repair enzyme access to the damaged DNA sites [7,9], and is involved in double strand break repair, where PAR recruits ATM, MRE11, as well as topoisomerase 1 [7]. Conversely, PARP can also contribute to cell death; for example, in the case of severe DNA damage, such as that resulting from ischemia, PARP1 hyperactivation triggers NAD^+^ and ATP depletion, ultimately leading to cell death via apoptosis or necrosis [7].

PARP inhibitors are a relatively recent class of targeted therapeutic agents that can promote synthetic lethality. Synthetic lethality is defined as cell death induction by the combined action of two factors that independently are not lethal; for example, in BRCA-mutated cells where the Homologous recombination (HR) pathway is defective, PARP inhibitors obstruct BER, leading to cell death [10]. PARP inhibitors target and inhibit PARP, and thereby the repair of SSB by BER, leading to severe double-strand breaks (DSB). HR-proficient cells can repair DSB originating from SSB, while HR-deficient cells undergo cell death [11].

Olaparib was the first clinically approved PARP inhibitor, specifically for the treatment of ovarian carcinoma, peritoneal carcinoma, metastatic pancreatic cancer, metastatic castration-resistant prostate cancer as well as HER-2 negative breast cancer [12]. After Olaparib, additional PARP inhibitors that were developed include Rucaparib, Niraparib as well as Talazoparib. Rucaparib is approved for the treatment of ovarian carcinomas, peritoneal carcinoma, and metastatic castration-resistant prostate cancer. Niraparib is also approved for ovarian carcinoma and peritoneal carcinoma treatment while Talazoparib is approved for advanced HER2-negative breast cancer treatment [12].

As is frequently the case with other chemotherapeutic agents [13], acquired resistance to PARP inhibitors has been reported, somewhat limiting their clinical utility. Molecular mechanisms that have been associated with the development of resistance to PARP inhibitors include up-regulation of the p-glycoprotein efflux pump, PARP overexpression as well as a shift in the BRCA mutational reading frame, which may explain why not all BRCA-mutated tumours are sensitive to PARP inhibition [7].

### Autophagy

Autophagy is a process that generally occurs under conditions of nutrient deprivation of other forms of cellular stress, wherein cytoplasmic components are provided to lysosomes for degradation, maintaining cellular homeostasis via the provision of a source of energy as well as metabolic intermediates [14]. This occurs through a series of sequential steps starting with formation of the phagophore, a double membrane structure that enfolds damaged cytoplasmic constituents [15]; the phagophore extends with cytoplasm engulfment, forming the autophagosome, which then fuses with lysosomes, resulting in autolysosome formation for bulk degradation [3,15]. This multistep process is closely regulated by several highly conserved autophagy (ATG) proteins as well as various cellular pathways including the phosphatidylinositol 3-kinase/mammalian target of rapamycin (PI3K/mTOR), extracellular signal-regulated kinase (Erk1/2) as well as AMP-activated protein kinase (AMPK) signaling pathways [16,17].

Unc-51-like kinase 1 (ULK1) plays a central role in autophagy initiation. Specifically, ULK1 responds to signals regarding the cell’s nutritional state, and is involved in recruitment and recycling of proteins to and from the phagophore assembly site that are required for autophagosome formation [18]. A variety of cellular stresses including hypoxia, oxidative stress, and starvation [3,19,20] activate ULK1 and trigger autophagy. A critical step in autophagic flux is the interaction between ULK1 and AMPK, which plays a vital role in directly promoting autophagy via the phosphorylation of various autophagy-related proteins including ULK1; in addition, this interaction regulates the expression of different autophagy-related genes downstream of transcription factors including transcription factor EB (TFEB), Forkhead box O3 (FOXO3), and Bromodomain-containing protein 4 (BRD4) [21]. This interaction between ULK1 and AMPK is regulated by mTOR, which acts as the major negative autophagy-regulating pathway. mTORC1 phosphorylates ULK1 at Ser757 to decrease ULK1 activity [22], disrupt ULK1-AMPK interaction, and prevent autophagy [23]. mTOR also phosphorylates and inhibits the nuclear translocation of a variety of transcription factors that are required for lysosomal biogenesis and the expression of autophagy related genes [24]. Another signalling pathway that controls the autophagic process is Erk1/2, which has been reported to act as a positive regulator of autophagy [25] by driving the expression of different autophagy and lysosomal related genes via activating TFEB, the master gene in lysosomal biogenesis [17,26].

**Figure 1:**
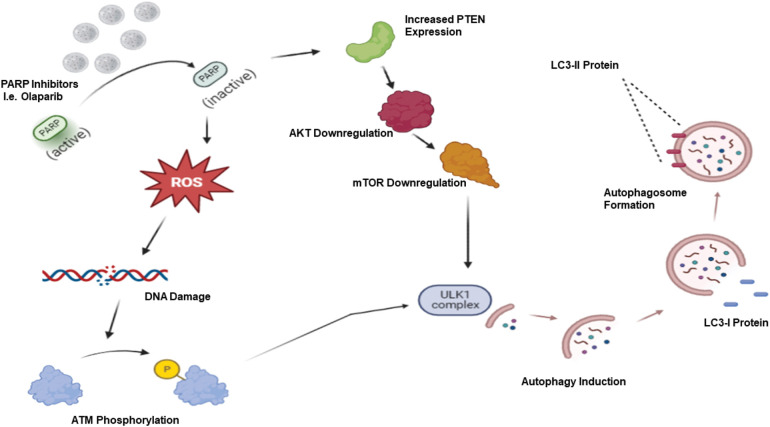
PARP inhibitor-induced autophagy. PARP inhibitors such as Olaparib generate reactive oxygen species (ROS), which results in a DNA damage response with increased ATM phosphorylation. Coupled with increased PTEN expression followed by AKT/mTOR downregulation, these events lead to ULK1 activation and autophagy induction.

Targeted therapy is considered a critically important advance in the field of cancer therapy, exhibiting lesser serious side effects than conventional non-selective chemotherapeutic agents. Different forms of targeted therapy that have been developed include monoclonal antibodies, small molecule inhibitors, antibody-drug conjugates, and immunotherapy [27]. Among these, PARP inhibitors have demonstrated efficacy, particularly for BRCA-related, high-grade ovarian cancer, BRCA-mutated breast cancer as well as triple negative breast cancer (TNBC). In our previous publications [2,3,28], we discussed the different functions of autophagy in cancer, focusing on the cytotoxic, cytoprotective, and non-protective forms (but omitting the cytostatic form, which has not been identified in most therapeutic modalities). Recently, the connection between autophagy and PARP inhibitors has attracted attention for the possibility of sensitizing different tumor models via autophagy targeting. Most studies show that PARP inhibitors trigger autophagy. For example, it is hypothesized that PARP inhibition via Olaparib leads to reactive oxygen species production which, in turn, generates a DNA damage response accompanied by increased γ-H2AX and ATM phosphorylation. Furthermore, Olaparib inactivates the AKT/mTOR pathway subsequent to increased PTEN expression, ultimately eliminating the inhibitory effect of mTOR on the ULK1 complex, leading to autophagy induction [29–31] (Fig. 1).

In this review, we will shed light on the functions of autophagy mediated by PARP inhibitors and evaluate the possible utility of targeting autophagy as adjuvant therapy to increase the effectiveness of these agents in the clinical setting.

## Olaparib and Autophagy

Olaparib was the first clinically approved PARP inhibitor. Olaparib was initially approved for *BRCA1/2* mutant ovarian and breast cancer patients, which is the basis for studies in these model systems that elaborated the association between Olaparib and autophagy. The therapeutic potential of targeting autophagy in combination with Olaparib has also been studied in various other malignancies including colon cancer, prostate cancer and leukemia.

### Breast cancer

Arun et al. [32] studied Olaparib efficacy and its relationship with autophagy in various breast cancer cell lines. Olaparib demonstrated significant growth inhibition in BRCA wild-type breast cancer cell lines with *BRCA1* *allelic loss* as compared to their BRCA wild-type counterparts. In addition, Olaparib inhibited the survival of *BRCA1* mutant (HCC-1937, MDA-MB-436, and SUM-149PT) as well as *BRCA2* mutant (HCC-1428) cell lines. Olaparib reduced cell viability while at the same time inducing autophagy in *BRCA1* mutant (SUM-149PT), *BRCA2* mutant (HCC-1428), as well as MDA-MB-231 cell lines with shRNA-mediated *BRCA1* or *BRCA2* knockdown. Autophagy was monitored based on increased acridine orange staining, LC3II protein expression, and autophagosome formation via transmission electron microscopy. Autophagy inhibition utilizing shRNA-mediated ATG5 knockdown *reduced* BRCA mutant cell lines sensitivity to Olaparib via a reduction of drug-induced mitochondrial degradation and apoptosis in the HCC-1428 cell line as the representative model. These observations reflect a *cytotoxic* role of autophagy in the BRCA mutant breast cancer cell lines.

Similarly, Min et al. [31] reported on the *cytotoxic* potential of Olaparib-induced autophagy when assessing the effects of combining Olaparib together with the histone deacetylase inhibitor, suberoylanilide hydroxamic acid (SAHA), in triple negative breast cancer (TNBC) cell lines. Olaparib in combination with SAHA showed synergistic cell growth inhibition and autophagic induction when compared to each drug alone, in MDA-MB-231, MDA-MB-157 and HCC1143 cell lines. The promotion of autophagy was demonstrated by increased LC3B and Beclin1 expression levels, as well as a GFP-tagged LC3 assay, indicative of a *cytotoxic* role for autophagy in these cell lines. The synergistic antitumor effect between Olaparib and SAHA was confirmed *in vivo* using a xenograft model of MDA-MB-231 cells grown in balb/c athymic nude mice. The combination’s synergistic effect was confirmed by a reduction in tumor volume, lowered Ki-67 expression, reduced AKT and ERK expression, as well as an increased apoptotic population [31].

Mechanistically, these authors focused on the synthetic lethality of PARP inhibition in DNA-repair deficient cells, as SAHA targets a key regulator of HR-related gene expression and nuclear assembly to further promote genomic instability and cell death. Moreover, they proposed that increased DNA damage and PTEN upregulation/autophagic induction contributes to the synergistic *cytotoxicity* of Olaparib and SAHA [31]. It was demonstrated that basal level of PTEN in PARP inhibitor-sensitive (MDA-MD-231 cell line) *vs*. PARP inhibitor-resistant cell lines (MDA-MB-468 cell line) impacted susceptibility to Olaparib and SAHA by increasing or decreasing autophagy through negative or positive regulation of AKT/mTOR expression, respectfully. PTEN phosphatase activity negatively regulates pAKT and pmTOR levels (Fig. 1), eliminating inhibitory effects on the ULK1 complex, and promotes autophagy. Unsurprisingly, shRNA PTEN knockdown increased AKT/mTOR expression while decreasing LC3B and Beclin-2 expression and GFP-LC3 punctate structures in PARP inhibitor-sensitive MDA-MB-231 cells. Conversely, transient overexpression of PTEN in resistant MDA-MB-468 cells resulted in an increase of LC3B and Beclin-1 expression, consistent with the promotion of autophagy [31].

It is important to note some limitations in the interpretation of aforementioned study [31] in that no pharmaceutical or genetic autophagy inhibition studies were performed to more conclusively identify the nature of the autophagy. Furthermore, given that PTEN can regulate both apoptosis and autophagy independently of one another, further targeted studies would be useful to narrow down whether increased cytotoxicity is the result of autophagy induced cell death or PTEN promotion of mitochondrial degradation.

Taking a slightly different direction with the same MDA-MB-468 TNBC cell line, Ren et al. [33] studied the combination of Olaparib with a novel ULK1/2 inhibitor, SBP-7455. As is generally the case, Olaparib induced autophagy, as confirmed by the mCherry-EGFP-LC3 assay, with an almost 30% increase in autophagic flux compared with the control. Furthermore, SBP-7455 was shown to effectively inhibit autophagic flux and promote cell death as confirmed by mCherry-GFP-LC3 and Annexin-V assays, respectively [33]. In addition, SBP-7455 specifically targeted early autophagosome formation by reducing downstream expression of Beclin1 Ser15 and VPS34 S249 [33]. Finally, SBP-7455 synergized with Olaparib in reducing the viability of the MDA-MB-468 cell line as compared to each drug alone, while suppressing Olaparib-induced autophagy as shown by the mCherry-GFP-LC3 assay. Therefore, in contrast to the previous findings, the data here are strongly indicative of a *cytoprotective* role played by autophagy [33]; however, as in the studies described above, this conclusion requires further validation by the incorporation of genetic approaches for autophagy inhibition.

Among the most recent studies performed with TNBC cancer models, Uddin et al. [34] studied Olaparib in BRCA wild-type SUM159-P and MDA-MB-468-P (HRD) TNBC cells and their Olaparib-resistant counterparts, SUM159-R and MDA-MB-468-R. Resistance in these cells was acquired through the inhibition of basal and induced PAR activity. Olaparib induced autophagy in both parental and resistant cell lines, as indicated by LC3I to LC3II conversion, while significantly inhibiting colony formation of SUM159-P and MDA-MB-468-P cells, supporting a *cytotoxic* function of autophagy. Combination treatment with Rapamycin, an autophagy inducer, and Olaparib synergistically inhibited both parental and Olaparib-resistant SUM159 and MDA468 cells growth, again consistent with *cytotoxic* autophagy, although in this case possibly due to Rapamycin. However, quite unexpectedly, enhanced growth inhibition was also observed when autophagy was inhibited by Chloroquine (CQ) in combination with Olaparib in a dose-dependent manner, evidence for *cytoprotective* autophagy. This apparent duality of cytotoxic and cytoprotective autophagy in the same model system could be attributed to toxicity of CQ in SUM159 cell lines, eliciting a general growth inhibitory effect. Again, the absence of genetic suppression of autophagy somewhat diminishes the rigor of any conclusions relating to cytoprotective autophagy.

Further proteomic analysis of PARP inhibitor-sensitive and resistant cell lines identified significant downregulation of basal Sequestosome 1 (p62/SQSTM1, indicated as p62) in both SUM159-R and MDA-MB-468-R cells by Olaparib. p62 interacts with LC3 and ubiquitin proteins to initiate autophagosome formation, and is subsequently engulfed by autophagosomes and degraded by autophagolysosomes [35]. Often considered a standard indication for autophagic flux, elevated p62 levels inversely correspond with low levels of autophagy. In Olaparib-free conditions, SUM159-R cells maintain high steady-state levels of autophagy with lower p62 levels compared to their parental counterpart, suggesting the active involvement of autophagy in Olaparib resistance [34]. Upon shRNA mediated knockdown of p62, which might be considered to reflect the promotion of autophagy, increased resistance to Olaparib was noted in both SUM159-P and SUM159-R cells. However, a putative connection made between autophagy and Olaparib through p62 expression levels is insufficient to conclusively distinguish between cytotoxic *vs*. cytoprotective roles of Olaparib induced autophagy. The potential off-target effects of modulation of p62 levels through NF-kB signaling, independent of autophagy, could influence drug sensitivity [36].

Taken together, the relationship between Olaparib and autophagy in breast cancer cell line models is somewhat inconsistent. Among the four studies analyzed, evidence for both cytotoxic and cytoprotective functions of autophagy were noted. Limitations to studies in TNBC cell lines include the heterogeneous nature of the broad subtype and consequent heterogeneous response to therapeutic treatments.

### Ovarian cancer

Shifting away from breast cancer models, a number of studies assessed the relationship between autophagy and Olaparib in ovarian cancer models to understand how the autophagic role can be manipulated in therapeutic treatments. Vescarelli et al. [37] reported that BRCA1-null UWB cell lines, with an underlying defective DNA damage response (DDRD) were the most sensitive to Olaparib, followed by BRCA1-restored UWB and BRCA-wt SKOV3 cell lines as shown by the MTT assay and clonogenic survival. Olaparib increased apoptosis in the BRCA1-null UWB cell line, while no significant apoptosis was detected in either *BRCA1* restored UWB or SKOV3 cell lines (based on multiple assays including Annexin A5 FITC/7AAD, cleaved caspase-3 and PARP1 expression levels). The lack of Olaparib-induced apoptosis in *BRCA1* restored UWB cells was accompanied by increased autophagy induction, as evident by a significant increase in the LC3-II:LC3-I ratio and degradation of p62. Subsequent inhibition of autophagy with CQ failed to sensitize *BRCA1* restored UWB cells, and, in fact, reduced the apoptotic markers, cleaved Caspase 3 and cleaved PARP1 expression, suggestive of *cytotoxic* autophagy [37]. Interestingly, there was no significant autophagy induction in either the BRCA1-null UWB or SKOV3 cell lines, thought to be a consequence of extensive autophagy-independent cell death.

Various drug combinations targeting upstream regulators of metabolic resources to optimize Olaparib treatment efficacy have been considered. Sui et al. [38] studied the possibility of combining Olaparib with the EGFR tyrosine kinase inhibitor, Erlotinib, in EGFR-overexpressing, *BRCA1/2* wild-type A2780 BALB/C nude mice xenograft models. The epidermal growth factor receptor (EGFR) has been proposed as a promising therapeutic target in ovarian cancer, as up to 70% of ovarian tumors are EGFR-positive. EGFR overexpression has been reported during ovarian cancer progression and correlates with a poor prognosis [39,40]. Olaparib combined with erlotinib showed significant antitumor activity as compared to each drug alone, but interestingly in the absence of a significant increase in apoptosis. Importantly, Olaparib combined with erlotinib showed greater autophagy induction than each drug alone, as confirmed by MDC staining, LC3II and Beclin 1 levels. A synergistic reduction in cell survival by the combination therapy appeared to be more dependent on autophagy than apoptosis Pharmacological autophagy inhibition using 3-methyladenine (3-MA) suppressed the combination antitumor activity, keying in on the dependency on autophagy over apoptosis and the *cytotoxic* role of autophagy [38]. Again, the absence of genetic autophagy inhibition strategies limits the confidence relating to conclusions as to autophagy function in this experimental system.

In contrast to the previous findings [38,37], Santiago-O’Farrill et al. [29] reported the opposite function of Olaparib-induced autophagy while investigating Olaparib effects in OVCAR8, HEY, A2780, and SKOV3 ovarian cancer cell lines. As would be anticipated from all the current literature, Olaparib induced autophagy in these cell lines as confirmed by an increased LC3II/LC3I ratio, autophagosome formation detected by transmission electron microscopy, elevated GFP-LC3 puncta, as well as mRFP-GFP-LC3 red puncta indicative of autophagic flux. Pharmacological autophagy inhibition using CQ, hydroxychloroquine (HCQ) and LYS05 resulted in a synergistic growth inhibition and increased apoptosis as shown by clonogenic survival assays, a sulforhodamine B (SRB) assay as well as Annexin-V flow cytometry. The same trend was also evident when autophagy was genetically inhibited using RNAi mediated knockdown of ATG5 or ATG7, with an enhancement of Olaparib mediated sensitivity, providing rigorous support for the *cytoprotective* role of Olaparib-mediated autophagy in these cell lines [29]. These findings were supported in both PH063 PDX and OVCAR8 derived xenografts, where tumor area and tumor weight were significantly lower in nu/nu mice treated with the combination of CQ + Olaparib as compared to Olaparib alone. Immunohistochemistry staining of tumor tissue showed a significant increase in LC3 protein with Olaparib, confirming autophagic induction following treatment [29].

Further analysis of the relationship between DNA damage and autophagy by this research group identified a partial dependency on ROS, ataxia-telangiectasia mutated (ATM) signaling, and PTEN signaling through the PI3K/AKT/mTOR pathway [29]. OVCAR8 and HEY cell lines treated with Olaparib experienced a dose-dependent increase in apoptosis, γ-H2AX nuclei accumulation, ATM and PTEN expression, G2/M cell cycle arrest, as well as ROS generation. Consistent with the findings by Min et al. [31], Santiago-O’Farrill et al. [29] reported that high-throughput antibody array highlighted the induction of autophagy through increased PTEN, decreased AKT Ser473, mTOR Ser4228, and PRAS40 expression in OVCAR8 cells following Olaparib treatment. Interestingly, combination treatments of antioxidants N-acetylcysteine (NAC) or melatonin with Olaparib decreased LC3II/LC3I ratios in both cell lines as compared to Olaparib alone, revealing a partial role played by ROS in autophagy induction. However, the destructive nature of ROS and broad sequestration of antioxidants makes it difficult to narrow its mechanism of action towards autophagy and, subsequently, its degree of effect on autophagic induction [29].

Although, autophagy *induction* following Olaparib administration is commonly observed, as noted in previous sections, Wang et al. [41] reported that Olaparib *reduced* the extent of autophagy and promoted apoptosis in A2780 and SKOV3 BRCA wild-type ovarian cancer cells as confirmed by decreased LC3I to LC3II conversion and increased expression of the pro-apoptotic Bax protein. Moreover, Bcl-2-associated athanogene 3 (BAG3) knockdown in combination with Olaparib produced a synergistic dose-dependent decrease in cell viability in both A2780 and SKOV3 cell lines; this sensitization was reduced when autophagy was promoted by rapamycin. Increased drug sensitivity in cells with reduced autophagy, that could be rescued by pharmacologic stimulation of autophagy, is indicative of *cytoprotective* autophagy, but in the broad sense of pro-survival function as a whole [41].

During the course of ovarian cancer, the epithelial cells detach from the extracellular matrix (ECM) and form spheroids to spread throughout the peritoneal cavity, leading to peritoneal metastasis, which is a primary characteristic of high-grade serous ovarian carcinoma (HGSOC); however, how these spheroids maintain their viability after ECM detachment has been largely unknown [42]. Recently, Lai’s laboratory [42] studied the relation between autophagy and metastasis using A2784 and SKOV3 ovarian cancer cell lines. In this work, SKOV3 spheroids displayed greater levels of basal autophagy compared to adherent cells independent of any treatments, as shown through the mRFP-GFP-LC3 assay. Consistent with Wang et al. [41] findings, Lai’s laboratory [42] showed that Olaparib treatment *reduced* autophagy in SKOV3 and A2780 adherent and spheroid cells, with a consequent decrease in cell viability. Interestingly, Olaparib treatment decreased autolysosome formation based on the mRFP-GFP-LC3 assay. Similar to the previous findings [41], Olaparib or bafilomycin A1 (Baf A1) inhibition of autophagy decreased cell viability, suggestive of a *cytoprotective* role for autophagy in these cells [42].

Mechanistically, a connection was proposed between autophagy, iron hemostasis and lipid metabolism, with SCD1 as a key modulator. Stearoyl coenzyme A desaturase 1 (SCD1) is an iron-containing lipogenic enzyme that aids in unsaturated fatty acid synthesis [43]. Both autophagy and iron levels were reported to be higher in spheroid than in adherent cells [42]. The elevation of iron was also prevalent in *in vivo* studies using SKOV3 and A2780 xenografts, or cells isolated from primary HGSOC tissue inoculated in BALB/c nude mice. *Autophagy reduction* as a consequence of Olaparib treatment or RB1CC1 knockdown, necessary for autophagosome formation, reduces the level of bioavailable iron by inducing iron sequestration/accumulation in lysosomes as shown by Prussian reagents and LAMP-1 stain. In the absence of iron, SCD1 is impaired, reducing lipid levels and viability. Interestingly, other pharmacological inhibitors of autophagy such as Baf A1 or CQ do not elicit the same inhibitory effect on SCD1 or reduce lipid levels in ovarian cancer spheroids. These findings argue for a direct involvement of autophagy in spheroid survival by promoting efficient iron homeostasis, SCD1 regulation and lipid metabolism [42].

The studies of Olaparib in ovarian cancer models not only provide evidence for different functional forms of autophagy but indicate that even with the same (A2780) cell line, Olaparib was reported to either promote [38] or reduce autophagy [41,42].

### Prostate cancer

Cahuzac et al. [44] studied Olaparib in the LNCa, C4-2B, as well as PC-3 prostate cancer cell lines. The LNCa and C4-2B cells were found to be relatively sensitive to Olaparib, while the PC-3 cell line was relatively resistant. Interestingly, the PC-3 cell line showed higher expression levels of Atg5 and the lipidated forms of LC3 A/B/LC3-II, suggesting that PC-3 cells have a higher level of basal autophagy than the other cell lines, a conclusion confirmed by the GFP/RFP tagged LC3-II assay. Importantly, they showed that genetic autophagy inhibition using CRISPR/Cas9–mediated Atg16L1 knockdown significantly sensitized LNCaP, C4-2B, as well as PC-3 cells to Olaparib, consistent with a *cytoprotective* role of autophagy. Although higher levels of autophagy promoted an Olaparib-resistant phenotype in the PC cell lines, the degree of cyto-protection appeared to vary based on the time of autophagy induction before or after Olaparib exposure. Cells pre-treated with rapamycin prior to Olaparib, thereby promoting elevated levels of basal autophagy, displayed a resistant phenotype not observed in cells treated with rapamycin after Olaparib.

Mechanistically, these investigators proposed that the varying degree of cytoprotection is related to the overall efficiency of DNA damage repair [44]. Similar to the proposed relationship described by Uddin et al. [34], autophagy activation by Rapamycin prior to Olaparib can be seen to act through p62 degradation to limit Olaparib’s effect on cell proliferation, cell cycle arrest, and DNA damage accumulation [44]. Rapamycin pre-treatment significantly reduced p62 levels in cells at a higher rate than those treated with rapamycin post-Olaparib. DNA damage repair is connected to p62 degradation through the negative regulator effect p62 has on filament A (FLNA) nuclear localization and its recruitment of Rad51 and *BRCA1* to double strand breaks. Therefore, rapamycin pre-treatment is considered to enhance HR in LNCaP and C42B cells, as reflected by increased Rad 51 and *BRCA1* foci, and decreased γH2AX. Interestingly, silencing of p62 via siRNA reversed the effect of autophagy inhibition in Atg16L1 LNCaP, C4-2B, and PC-3 knockout cells, producing a similar resistant phenotype to cells with elevated autophagy and HR efficiency.

### Hematologic malignancies

In studies involving hematological malignancies, Blomhoff’s lab has reported that cAMP activating factors present in the bone marrow render B-cell precursor acute lymphoblastic leukemia (ALL) cells less sensitive to DNA damage–induced apoptosis through autophagy induction and suppression of p53 [45]. Within the normal tumor microenvironment, B-cell precursor acute lymphoblastic leukemia (ALL) are exposed to cAMP-stimulating factors in the bone marrow [46]. Utilizing the same model of drug interaction *in-vitro*, Richartz et al. [47] studied the impact of Olaparib on cAMP-induced autophagy. Olaparib pre-treatment followed by exposure to forskolin, an adenylyl cyclase activator, alone or in combination with ionizing radiation, increased apoptosis while reducing levels of cAMP-induced autophagy in REH cells, as confirmed through reduced LC3II WB expression and CYTO-ID autophagy vesicle staining. Radiation-resistant REH cells are resensitized following Olaparib treatment through the inhibition of cAMP-induced autophagy. These findings were upheld in NSG mice implanted with REH cell and primary leukemic xenograft models irradiated in the presence or absence of Olaparib, where tumor burden quantified through luciferase-EGFP and $CD19^{+}CD10^{+}$ was reduced by Olaparib. Here the autophagy appeared to be *cytoprotective* in function.

### Colon cancer and lung cancer

Studies in our own laboratory evaluated the PARP inhibitors, Olaparib and Niraparib, in combination with radiation using HCT116 colon cancer cells that were either wild-type, DNA proficient, or HCT116 ligase IV deficient with impaired nonhomologous end joining repair [48]. Both PARP inhibitors promoted sensitivity to radiation by increasing the extent of the radiation-mediated DNA damage in the absence of a significant change in the level of apoptosis. In fact, the combination treatment increased the extent of senescence, as evidenced by the higher intensity of β-galactosidase staining and greater extent of growth arrest at the G2/M phase as compared to the controls [48]. Importantly, Niraparib or Olaparib in combination with radiation increased levels of autophagy to a greater extent than radiation alone. Autophagy inhibition via shRNA mediated ATG5 and ATG7-targeting or the pharmacological autophagy inhibitor, CQ, did not affect the Niraparib or Olaparib-induced radiosensitization, indicative of *nonprotective* autophagy [48]. Similar evidence for *nonprotective* autophagy was generated in H460 non-small cell lung cancer cells where autophagy inhibition, genetically or pharmacologically, did not affect the PARP inhibition-mediated sensitization to radiation [48].

Taken together, it is evident that the nature of Olaparib-induced autophagy is not consistent, nor can it be predicted in different tumor models.

## Niraparib and Autophagy

### Laryngeal carcinoma

Ji et al. [49] studied the association between Niraparib and autophagy using TU212 and TU686 laryngeal squamous cell carcinoma (LSCC) cell lines. Niraparib treatment induced autophagy, as confirmed by autophagosome accumulation in the TU212 cell line using transmission electron microscopy, high green florescence of CYTO-ID (autophagy-specific green dye), indicating LC3-II protein expression on the autophagosomal membrane, as well as dose and time dependent elevation in LC3-II and a reduction in p62/SQSTM1 expression levels in both TU212 and TU686 cell lines. In addition, Niraparib reduced the phosphorylation of Akt, mTOR, and 4E-BP1 in a dose-dependent manner while increasing the phosphorylation of Erk1/2, suggesting that Niraparib-mediated autophagy is regulated by Akt/mTOR and Erk1/2 signalling pathways [49]. Autophagy inhibition using the standard pharmacological approaches with CQ remarkably increased Niraparib cytotoxicity in the Tu686 and Tu212 cell lines, indicating a *cytoprotective* role for autophagy. However, as noted throughout this review, autophagy inhibition utilizing CQ alone is considered a limitation of efforts to define autophagy function.

Mechanistically, Ji et al. [49] attempted to explain how Niraparib in combination with CQ resulted in a significantly greater cytotoxicity than each drug alone. Their studies showed that the combination caused a reduction in Cyclin D expression levels, indicating that suppression of autophagy decelerated the LSCC cell cycle. In addition, the combination treatment induced DNA lesions, as evidenced by a significant increase in γH2AX foci, which was confirmed by increased γH2AX expression by western blotting. Interestingly, Niraparib triggered the phosphorylation of Chk1, causing HR initiation, whereas suppression of autophagy via CQ decreased the phosphorylated Chk1 levels, via causing a progressive increase in the proteasomal activity in a time-dependent manner in both LSCC cell lines, and thereby impairing HR. These experiments suggested that HR mediated by Niraparib exposure was impaired by autophagy inhibition [49].

### Hepatocellular carcinoma

Consistent with the findings by Ji et al. [49], Zai et al. [25] also reported that Niraparib induced autophagy in Huh7 and HepG2 hepatocellular carcinoma cell lines, as confirmed by the cytoplasmic aggregation of double-membrane vesicles, Cyto-ID fluorescence accumulation as well as the marked increase in LC3-II protein expression in a time- and dose-dependent manner. Furthermore, they showed that Niraparib not only initiates autophagy but also promotes the completion of autophagic flux in the Huh7 and HepG2 cells, as evidenced by Cyto-ID combination staining with lysosome-specific Lyso-Tracker dye. Additionally, co- treatment with the late-stage inhibitor of autophagy, CQ, increased the expression of LC3-II and p62 [25]. This work also supported the findings by Ji et al. [49], as Niraparib significantly decreased mTOR phosphorylation in a time- and dose-dependent manner, inhibited Akt phosphorylation as well as caused a reduction in 4EBP1 phosphorylation. Moreover, Niraparib significantly upregulated the phosphorylation of Erk1/2 in a time- and dose dependent manner in both Hun7 and HepG2 cell lines [25].

Autophagy inhibition using the pharmacological inhibitor, bafilomycin A1 (Baf A1), enhanced Niraparib-induced cytotoxicity, suggesting the *cytoprotective* role played by Niraparib-induced autophagy in both Hun7 and HepG2 cell lines. Similarly, the combination of Niraparib and CQ showed enhanced cytotoxicity (and apoptosis) in both Huh7 and HepG2 cells. The combination treatment also caused significant suppression of Huh7 and HepG2 colony formation as compared to the controls.

Consistent with the Ji et al. [49] results, Zai et al. [25] reported that the Niraparib and CQ combination synergistically increased γ-H2AX foci, while decreasing the expression of RAD51, an HR repair machinery marker, as well as inhibiting *BRCA1* expression, indicating increased double-strand breaks and compromised DNA repair efficiency in both Huh7 and HepG2 cell lines. Interestingly, they showed that the synergistic effect of the combination was independent of p53, since this synergy was evident in both p53-mutant Huh7 as well as p53-null Hep3B cell lines. Moreover, they confirmed the synergy between CQ and Niraparib *in vivo* using the Huh7 xenograft mouse models. More specifically, Niraparib in combination with CQ showed a synergistic antitumor efficacy as compared to either drug alone without apparent loss in body weight, or systemic toxicity [25], suggesting that the autophagy mediated by Niraparib played a *cytoprotective* role; however, as in many cases, genetic autophagy inhibition approaches were not included. What is, however, unique in this study, is that the synergism between CQ and Niraparib was shown to be specific to the tumour cells, as no synergistic effect was observed in the non-transformed HepaRG cells [25].

### Ovarian and breast cancer

Booth et al. [50] reported the molecular steps whereby Niraparib induced autophagy in spiky ovarian cancer cells and BT474 mammary cancer cells. Niraparib initiated DNA damage response signalling by inducing ATM activation, resulting in AMPK and ULK1 stimulation, together with mTOR inhibition, ultimately resulting in enhanced ATG13 S318 phosphorylation, promoting autophagosome formation. Then, over time, the levels of autophagosomes declined while the levels of autolysosome increased, suggesting on going autophagic flux. Importantly, Niraparib-mediated cytotoxicity was significantly reduced via siRNA mediated Beclin1 or ATG5 knock down [50], indicating the *cytotoxic* role played by Niraparib-induced autophagy in both ovarian cancer cells and mammary cancer cells.

Recently, Wang et al. [51] studied the effect of combining Niraparib with short-term starvation (STS), which is a classical approach for promoting autophagy, using SKOV3 and A2780 ovarian cancer cell lines. Pre-treatment with STS synergistically enhanced Niraparib-mediated cytotoxicity. Importantly, Niraparib showed the characteristics of autophagy induction, including p62 degradation as well as LC3II accumulation. Interestingly, autophagy was intensified upon combining STS with Niraparib, based on a further reduction in LC3II and p62/SQSTM1 levels, respectively. Utilizing the mRFP-GFP-LC3 assay to follow up the progression of autophagy in SKOV3 and A2780 cells, these investigators reported a mass of red puncta with Niraparib alone, indicating the aggregation of autophagosomes as well as autolysosomes, which was further intensified when Niraparib was combined with STS [51]. While these outcomes could be suggestive of a *cytotoxic* role for Nirabarib-mediated autophagy, this conclusion must be withheld in the absence of studies of autophagy inhibition with pharmacologic and genetic approaches.

### Colon and lung cancer

As mentioned previously, our laboratory studied the PARP inhibitor, Niraparib, in combination with radiation using H460 non-small cell lung cancer and HCT116 colon cancer cell lines [48]. Niraparib in combination with radiation increased the extent of autophagy over and above that for radiation alone, as confirmed by the screening of autophagic cell number by Flow cytometry. However, there was no significant effect of either pharmacological or genetic autophagy inhibition, indicating the *non-protective* role of autophagy in these cell lines.

As was the case with Olaparib, the nature and function of the autophagy induced by Niraparib varied among the experimental systems investigated.

## Talazoparib and Autophagy

### Chronic myeloid leukemia

Liu et al. [52] studied the relationship between talazoparib and autophagy using pediatric chronic myeloid leukemia cells (CML). Talazoparib triggered autophagy, as shown by marked autophagosomes accumulation, increased Cyto-ID florescence, increased LC3-II levels as well as reduced levels of p62. Autophagy inhibition with CQ significantly potentiated talazoparib induced cytotoxicity in the CML cells [52], indicating a *cytoprotective* role played by talazoparib mediated autophagy. This *cytoprotective* role was confirmed in studies where genetic inhibition of autophagy by siRNA- mediated *ATG5* knockdown also enhanced talazoparib induced cytotoxicity in CML cells. The synergistic effect of combining autophagy inhibition with talazoparib was further confirmed *in vivo* using the CML PDX model where CQ increased the antitumor effect of talazoparib [52].

### Breast cancer

Pai Bellare et al. [53] also investigated the possibility of targeting autophagy as an adjuvant to talazoparib therapy to overcome acquired resistance to talazoparib. Talazoparib alone minimally affected the viability of both MCF-7 and MDA-MB-231 breast cancer cells, with little evidence for apoptosis. Likewise, talazoparib failed to significantly reduce tumor burden using BRCA-WT-SCID-mice xenografts [53]. However, as would be anticipated, BRCA1-KO MCF-7 cells showed a high sensitivity to talazoparib. Talazoparib induced autophagy in the MCF-7 cells based on the significant enhancement in ATG3, ATG5, ATG7 and ATG16L1 levels, significant elevation in EGFP-LC3 puncta levels as well as p62 colocalization with LC3 as evaluated by immunofluorescence microscopy. Talazoparib also induced autophagy in the MDA-MB-231 cell line, as evidenced by p62 colocalization with LC3 as well as the elevation in endogenous LC3B puncta using immunofluorescence microscopy. These investigators further confirmed that talazoparib enhanced autophagic flux in the MCF-7 breast cancer cell line by using the lysosomal acidification and late-stage autophagy inhibitor, Bafilomycin A1, which showed LC3-II accumulation in a time dependent manner as compared to talazoparib alone; in addition, p62/SQSTM1 degradation observed with talazoparib was blocked in the presence of BafA1. A similar trend was observed with another late stage autophagy inhibitor, CQ, which blocked talazoparib-induced clearance of p62 in the MCF-7 cells [53]. The enhanced autophagic flux was further confirmed in both BRCA1-WT and BRCA1-KO MCF-7 cells using the mRFP-EGFP-LC3 assay both in the absence and presence of CQ [53], in which BRCA1-WT MCF-7 cells showed enhanced red fluorescence, indicating acidification of autophagolysosomes, and yellow fluorescence (mixture of red and green), indicating mature autophagosome punctae upon Talazoparib treatment. Similar results were also obtained in BRCA1-KO MCF-7 cells in response to Talazoparib alone. Talazoparib-mediated quenching of green fluorescence was abrogated upon CQ treatment, indicating a robust autophagosome formation as well as autophagic flux in both BRCA1-WT and BRCA1-KO MCF-7 cell lines.

Having clearly established autophagy induction and autophagic flux by multiple assays, the effects of autophagy inhibition were then investigated pharmacologically using CQ, which was shown to significantly sensitize MCF-7 and MDA-MB-231 cell lines to talazoparib, based on apoptosis induction as well as reduced colony formation. A similar trend was evident with another autophagy inhibitor, 3-methyl adenine, with enhanced sensitivity to talazoparib [53]. Mechanistically, autophagy inhibition by CQ was shown to stimulate deleterious NHEJ mediated DSB (double strands breaks)-repair, ultimately causing extensive genomic instability as well as mitotic catastrophe. The BRCA1-KO cell line was more sensitive to talazoparib than when combined with CQ, which is correlated with known HR-deficiency in these cells, indicating that here autophagy inhibition does not appear to play a significant role.

The *cytoprotectiv*e role of autophagy was further confirmed using a genetic approach by generating MCF-7 cells lacking ATG5, p62 or LAMP1 using CRISPR-Cas9 based technology. Genetic autophagy inhibition in combination with talazoparib resulted in significant sensitization in a clonogenic survival assay [53], indicating the *cytoprotective* role mediated by autophagy. Interestingly, BECN1 knockout did not sensitize MCF-7 cells to talazoparib, indicating that BECN1 is unessential for talazoparib-induced autophagy in MCF-7 breast cancer cells [53].

Additional studies investigated the impact of combining CQ with talazoparib in T47-D, MDA-MB-453 and SKBR-3 breast cancer cell lines; here the combination showed a significant sensitization, confirming the *cytoprotective* role played by talazoparib induced autophagy. The *in vitro* effect of the combination of talaxoparib with CQ was also confirmed *in vivo* using BRCA-WT-SCID-mice xenografts, where a reduction in tumor volume and growth rate was greater than for each drug alone with no significant toxicity [53]. Together, these results delineate the *cytoprotective* role played by talazoparib induced autophagy in *HR-proficient* breast cancer cell lines.

Recently, Pai Bellare et al. [54] studied the potential of combining talazoparib with the natural molecule, resveratrol, in different breast cancer cell lines. Talazoparib combined with resveratrol produced a meaningful reduction in cell viability of MCF-7 and MDA-MB-231 breast cancer cell lines in both an MTT assay and a clonogenic survival assay; in addition, the drug combination caused a significant increase in the apoptotic population over that induced by each drug alone [54]. Importantly, the association between the combination of talazoparib and resveratrol with autophagy was also investigated. By utilizing an EGFP-LC3 assay, it was shown that that the number of puncta was high in MCF-7 cells with each drug alone as well as in the combination treatment, indicating autophagosome formation, which was confirmed by a reduction in p62 levels. In addition, ATG5 and ATG7 levels were increased in MCF-7 cells treated with either resveratrol alone or the combination. ATG5 and ATG7 levels were also enhanced with each drug alone as well as the combination in MDA-MB-231 cells, indicating autophagic induction which also confirmed by a reduction in p62 levels [54]. Interestingly, the LC3B-II/I ratio was decreased with talazoparib alone, but was enhanced with resveratrol alone and combination treatment of talazoparib plus resveratrol. These results suggested to these investigators that although resveratrol induced autophagosome formation, it hampered the fusion of autophagosomes with lysosomes, a conclusion that was confirmed by the mRFP-EGFP-LC3 assay. Specifically, enhanced red puncta (RFP^+^ and GFP^-^) was observed with talazoparib alone, indicating an effective autophagic flux with autophagosome and lysosome fusion; on the other hand, resveratrol treatment enhanced the yellow puncta (RFP^+^ and GFP^+^) levels. The yellow puncta were increased markedly in breast cancer cells with the resveratrol and talazoparib combination, indicating suppression of the fusion between autophagosomes and lysosomes and a defective autophagic flux. Moreover, there was a significant increase in lysosomal membrane permeabilization (LMP) with resveratrol treatment which further intensified with the talazoparib and resveratrol combination treatment using AO staining [54]. These data suggested that talazoparib effectively induced autophagosome formation and autophagy flux, while resveratrol delayed/inhibited the autophagy flux by targeting the lysosomal integrity [54], suggestive of the *cytoprotective* role of autophagy. However, confirmation of this conclusion awaits pharmacological and genetic autophagy inhibition studies.

Collectively, these results showed a greater tendency toward the cytoprotective role of talazoparib mediated autophagy and highlight the possibility of utilizing autophagy inhibition approach as adjuvant therapy with talazoparib in the clinical setting; however, further research is indicated, particularly with regard to the off-target effects of autophagy inhibitors as well as the potential impact on normal tissue function.

## Rucaparib and Autophagy

Despite an increasing number of clinical trials investigating the potential use of rucaparib in different malignancies and its accelerated approval for treatment of *BRCA1/2* mutant metastatic castration-resistant prostate cancers [55], there is little information in the literature on the autophagic potential of rucaparib and its subsequent influence on pharmacological interactions when used in combination with other drugs.

Porcelli et al. [56] studied rucaparib effects in the MiaPaCa-2 pancreatic cell lines. Rucaparib induced autophagy when combined with IR as shown through LC3-positive autophagosome generation without apoptosis using the MiaPaCa-2 cell line. However, these findings are insufficient for determining the specific role of autophagy played in these cells.

## Conclusions

The clinically approved PARP inhibitors have shown efficacy in the clinical setting in the treatment of various solid and hematologic malignancies. However, as with the case of other chemotherapeutic agents, the development of drug resistance often limits their clinical utility. Autophagy targeting that induced by PARP inhibition does have the potential to increase PARP efficacy and overcome acquired resistance, but only if the autophagy is cytoprotective in function. As summarized in Table 1, although the *cytoprotective* function is often observed, there is extensive evidence for a *cytotoxic* function, with a limited number of reports identifying a *non-protective* role. Furthermore, while the majority of the studies cited in this review support the conclusion that PARP inhibitors promote autophagy; a limited number reported that autophagy was suppressed. The most promising evidence for *cytoprotective* autophagy appears to be derived from the studies with talazoparib, which could lead to translation into clinical settings.

**Table 1:** Different functions of autophagy in response to PARP inhibitors

Agent	Cell lines/Cancer type	Autophagy role	References
Olaparib	HCC-1428 *BRCA1* mutant breast cancer cell line	cytotoxic	[32]
	MDA-MB-231, MDA-MB-157 and HCC1143 TNBC cell lines	cytotoxic	[31]
	MDA-MB-468TNBC cell line	cytoprotective	[33]
	SUM159 and MDA-MB-468; as well as their Olaparib-resistant counterparts, SUM159-R and MDA-MB-468-R TNBC cell lines	Nonprotective	[34]
	*BRCA1* null UWB, and *BRCA1* restored UWB ovarian cancer cell lines	Non-protective with *BRCA1* restored UWB	[37]
	A2780 (BRCA wild type) ovarian cancer cell line	cytotoxic	[38]
	OVCAR8, HEY, A2780 and SKOV3 ovarian cancer cell lines	cytoprotective	[29]
	A2780 and SKOV3 (BRCA wild type) ovarian cancer cell line	cytoprotective	[41]
	A2780 and SKOV3 ovarian cancer cell line	cytoprotective	[42]
	LNCaP and C4-2B and PC-3 prostate cancer cell lines	cytoprotective	[44]
	REH B-cell precursor acute lymphoblastic leukemia (ALL) cell line	cytoprotective	[47]
	H460 non-small cell lung cancer and HCT116 colon cancer cell lines	nonprotective	[48]
Niraparib	TU212 and TU686 laryngeal squamous cell carcinoma (LSCC) cell lines	cytoprotective	[49]
	Huh7 and HepG2 hepatocellular carcinoma cell lines	cytoprotective	[25]
	spiky ovarian cancer cells and BT474 mammary cancer cells	cytotoxic	[50]
	SKOV3 and A2780 ovarian cancer cell lines	cytotoxic	[51]
	H460 non-small cell lung cancer and HCT116 colon cancer cell lines	non-protective	[48]
Talazoparib	pediatric chronic myeloid leukemia cells	cytoprotective	[52]
	MCF-7, BRCA1-KO MCF-7, MDA-MB-231, T47-D, MDA-MB-453 and SKBR-3 breast cancer cell line	Cytoprotective except for BRCA1-KO MCF-7, showed non protective	[53]
	MCF-7 and MDA-MB-231 breast cancer cell lines	cytoprotective	[54]
Rucaparib	MiaPaCa-2 and Capan-1	cytotoxic	[56]
